# AI enhanced collaborative human-machine interactions for home-based telerehabilitation

**DOI:** 10.1177/20556683231156788

**Published:** 2023-03-20

**Authors:** Hoang H Le, Martin J Loomes, Rui CV Loureiro

**Affiliations:** 1 Wellcome/EPSRC Centre for Interventional and Surgical Science (WEISS), 4919 University College London, London, UK; 2 School of Science and Technology, Middlesex University, London, UK; 3 Royal National Orthopaedic Hospital, 4919 University College London, London, UK

**Keywords:** collaborative rehabilitation, engagement, haptic device, long-short term memory, motivation, nonlinear autoregressive models with exogenous input, social interaction, telerehabilitation, virtual reality

## Abstract

The use of robots in a telerehabilitation paradigm could facilitate the delivery of rehabilitation on demand while reducing transportation time and cost. As a result, it helps to motivate patients to exercise frequently in a more comfortable home environment. However, for such a paradigm to work, it is essential that the robustness of the system is not compromised due to network latency, jitter, and delay of the internet. This paper proposes a solution to data loss compensation to maintain the quality of the interaction between the user and the system. Data collected from a well-defined collaborative task using a virtual reality (VR) environment was used to train a robotic system to adapt to the users’ behaviour. The proposed approach uses nonlinear autoregressive models with exogenous input (NARX) and long-short term memory (LSTM) neural networks to smooth out the interaction between the user and the predicted movements generated from the system. LSTM neural networks are shown to learn to act like an actual human. The results from this paper have shown that, with an appropriate training method, the artificial predictor can perform very well by allowing the predictor to complete the task within 25 s versus 23 s when executed by the human.

## Introduction

Telerehabilitation robotics has grown remarkably in the past few years. It can provide intensive training to people with special needs remotely while facilitating therapists to observe the process.^1–4^ Telerehabilitation robotics is a promising solution supporting routine care, which can help to transform face-to-face and one-on-one treatment sessions. These sessions require intensive human resources and are restricted to some specialised care centres to treatments that are technology-based (less human involvement) and easy to access remotely from anywhere. However, some limitations, such as network latency, jitter, and internet delay, can negatively affect user experience and the quality of the treatment session. Moreover, the lack of social interaction since all treatments are performed over the internet can reduce patients’ motivation. As a result, these limitations make it very difficult to deliver an efficient recovery plan.^1,2^

Carignan et al.^3^ defined the major types of telerehabilitation interactions as: (i) unilateral: patient and therapy are examined with a time-delay; (ii) interactive bilateral: patient and therapist communicate through a virtual environment (e.g., video, virtual, and augmented reality) but without direct force-feedback in either direction; (iii) cooperative bilateral: therapist and patient communicate directly with each other, remotely but with video, force, and kinesthetics feedback.

A distributed VR-haptic-based system working in a shared virtual environment could enable two or more users to do the same task in remote locations. Nevertheless, the transparency of such a system is compromised by network issues that occur during long-distance communications, such as the loss of data packets or time delays.^4^ Psychologists have investigated delayed feedback’s effects on task performance since the 1960s. Kalmus et al.^5^ analysed the handwriting transmitted over a network and found that the delayed visual feedback increased completion time and errors made. A study conducted by Sheridan and Ferrell^6^ using master-slave robot arms also pointed out that visual latency was responsible for decreasing the performance of manipulation tasks.

Initial studies regarding delayed virtual feedback in collaborative virtual environments (CVEs) showed that the impact of the delay varied according to the difficulty of the task; therefore, it is impossible to pick a particular number as a threshold for the delay.^7,8^ For instance, Vaghi et al.^9^ performed a study of a collaborative virtual ball game where two players must hit a virtual ball into their opponent’s goal. The study provided qualitative evidence that the game could be played smoothly with a delay of 150 ms. However, with the increasing delay, it became harder to play and was almost impossible to continue when reaching a time delay of 500 ms. A study of a telerobotic surgery system conducted by Kim et al.^10^ showed that the performance was not affected until the delay reached over 250 ms. Besides, when the delay was around 400 ms, the operators found it more challenging to perform the task continuously.

Although the understanding of tolerable ranges of visual delay is still vague, it is evident that delayed visual feedback affects task performance in terms of increasing time taken to finish the task and error rates. A similar picture has been found with a delay in haptic feedback. However, the haptic delay tends to be more sensitive than visual latency; hence, its impact on performance is more significant. For example, a study revealed that the errors started to rise from haptic delays of 25 ms, while it only happened from visual delays of 50 ms.^7^

Recent studies have focused on compensating harmful effects due to haptic/physical delays caused by data loss via network environments. For example, Zhang et al.^11^ introduced a torque-limiter mechanism for their telerehabilitation system: whenever the interaction torque surpasses the predefined threshold, the torque-limiter will force the device to move freely regardless of its previous positions. On the other hand, Meli et al.^12^ proposed an approach to exclude force feedback data and only used position data for synchronisation between the client and server. The data loss could then be predicted with basic motion compensation. This approach helped to compensate for data transmission delays and, thus, facilitated activity completion without significant problems.

This paper proposes a different approach to train the system to predict user’s interactions in a VR-haptic based collaborative task performed by two participants. We employ two well-known methods: Nonlinear Autoregressive models with eXogenous input (NARX) using the Levenberg – Marquardt algorithm as a training algorithm and deep learning using a Long Short-Term Memory (LSTM) neural network. Nine datasets have been used for the training, and one dataset for the validation of trained networks.

## Background

### Delay concealment methods

Dealing with network impairments has been a crucial challenge for the field of robotic teleoperation for several decades. Several methods have been directed towards reducing the effect of network delay, however each method has its limitations thus only using one method alone still does not deliver the desired result. The four most common techniques that have been used widely in the field include:

Method 1 - Predictor:^13,14^ Usually, when transferring data via a network, the client side will keep track of the sequence of a number of packets it sends to the server. The server then sends back an acknowledgement flag to the client for each packet received. By doing it, the system can easily detect a packet loss that will be sent to the predictor unit. This unit is developed using a predictor algorithm which predicts the missing data based on previous received packets or human movement model such as minimum jerk theory. The remote client will then render the haptic feedback using the given result.

Positives: This method significantly reduces the delay caused by the network’s limitation. In addition, the predictor algorithm could be changed or optimised to give a better result.

Negatives: This method will cause a deviation between the receiver and the source. The predicted packet is always different from the original value. As a result, it may lead to incorrect interaction between users as well as raising unexpected safety issues.

Method 2 - Synchronisation Control Schemes:^15,16^ The server and each client have their delay synchronisation modules. These modules will work together to buffer incoming data, thus delaying haptic rendering at each client until all clients are synchronised.

Positives: Since all clients can obtain the same haptic display as the server without jolting and buzzing, the outcome force feedback is reliable, ensuring operational safety.

Negatives: This method highly depends on determining incoming data’s optimum buffer size, which could be another difficult challenge.

For instance, if the buffer size is small, haptic data will be lost and causes an unreliable output. On the other hand, if the size is too big, it will add an unnecessary system delay which slows down the response from the server to the client.

Method 3 - Data Compression:^17^ This algorithm divides haptic data streams into subsets based on human haptic perception. These data subsets will be reduced using a geometric distance approach. Each data subset is also fitted by a quadratic curve to improve approximation precision, and only coefficients of those quadratic curves will be sent to the destination instead of the original haptic data.

Positives: A large amount of haptic data may be reduced using this technique. Hence, it is extremely useful for a system that transmits voluminous haptic data. Moreover, it can be combined with other methods, such as a predictor, to eliminate the latency even more.

Negatives: This method is not very useful for transmitting a small amount of haptic data. In addition, using this method without combining with other techniques does not help resolve the delay caused by network limitations.

Method 4 - Multiple protocols:^18^ This method is a combination of multiple protocols such as the Synchronous Collaboration Transport Protocol (SCTP), the Selective Reliable Transmission Protocol (SRTP), the Reliable Multicast Transport Protocol (RMTP) and the Scalable Reliable Multicast (SRM) in a single system. The combined protocol has a multicast tree to avoid congestion and delay issues. It also ensures data reliability using multi modes of transmission.

Positives: By combining multiple protocols, this method can take advantage of the best features of different protocols have different features. As a result, the data transmission is reliable, the delay is minimised, congestion is avoidable, and synchronisation is achievable.

Negatives: Although this method seems ideal for dealing with network impairments, it still requires the multicast tree algorithm to work reliably, which is also a difficult challenge.

### NARX networks

Artificial neural networks (ANNs) have been used for various applications such as time series predictions, classification, recognition, optimisation, etc.^10,19–22^; ANN models are particularly beneficial for time-series predictions with noisy and nonlinear data. They usually outperform other standard linear techniques, e.g., Box-Jenkin models^23^ for such systems thanks to their capability of nonlinear mapping of m-dimensional inputs onto n-dimensional outputs while the relationship between the inputs and outputs are unknown^24^ and better robustness to noise.^25^

Nonlinear autoregressive model with exogenous inputs (NARX) is a well-known subclass of recurrent dynamic neural architectures. NARX networks have been proven to be computationally powerful in theory^26^ and a good predictor for time series.^27–29^

The NARX network, described by equation (1), predicts a time series *Z* at time *t* using as regressors the last *p* values of an external variable *U* and the last *p* values of the series itself. The nonlinear function *f* represents a feedforward network architecture and its weights. The input layer is usually known as the time window.

(1) Z(t)=f(U(t−1)…U(t−p)…Z(t−1),…,Z(t−p))+e(t)

The Levenberg-Marquardt (LM) algorithm is one of the most well-known algorithms for optimisation. The LM results in most problems are usually significantly better than simple gradient or other conjugate gradient methods.^30^ LM is a combination of vanilla gradient descent and Gauss-Newton iteration.

The Levenberg–Marquardt algorithm (provided by MATLAB neural network toolbox) has been applied to adjust the weights of the ANNs. The algorithm is presented as follows:

(2) $$w_{k}=w_{k+1}+∆w$$

(3) $$∆w={\left[J_{k}^{T}J_{k}+\eta I\right]}^{-1}J_{k}e_{k}$$

(4) $$e_{k}=r_{k}-z_{k}$$

Where *w* is the weight vector, *∆𝑤* is the difference between the weight vectors, *k* is the index of iterations*, J* is the Jacobian matrix that contains the first derivatives of the network errors with respect to the weight, *η* is a scale parameter, *I* is the identity matrix, *r* is a vector of the reference motion, *z* is a vector of the estimated motion, and *e* is a vector of network errors.

Positives: NARX networks converge much faster, need less training (lower training cycles), and are more effective (better gradient descent) than other networks. Moreover, they generalise better and thus can be applied in any nonlinear dynamical and time series system.

Negatives: NARX networks, similar to other gradient-based networks, have an issue called “vanishing gradient”, which has limitations in learning long-term dependencies and optimising embedded memory. As a result, having optimal input, output, and the number of neurons is very difficult, affecting NARX networks’ performance.

### LSTM neural networks

A recurrent neural network (RNN) is a class of neural networks which is derived from feedforward networks. While in a feedforward network, information can only move in one direction, a RNN can allow information to flow through a cycle as a loop. This looping mechanism with its internal memory makes the RNN very good at predicting sequential data since it can consider the inputs from both current and previous steps.

By the late 1980s, several pieces of research^31–33^ had pointed out that a backpropagation algorithm is complicated to be applied to train traditional RNNs. The primary reason has been identified by Hochreiter^34^ known as the long-time lag problem: computed errors from the backpropagation algorithm are either quickly shrunk or exploded (growing out of bounds). Supervised Long Short-Term Memory (LSTM) RNNs have been introduced^35–37^ to overcome this problem.

A LSTM network has a memory cell that remembers information from previous timesteps. It also has three gates (input, forget and output gate) that determine (by using sigmoid function) which information is allowed to pass through the cell state (input gate), stored or deleted (forget gate), and selected for the output (output gate). The equations for gates in a LSTM network are presented as follows:

(5) $$i_{t}={\sigma (w}_{i}\left[h_{t-1},x_{t}\right]+b_{i})$$

(6) $$f_{t}={\sigma (w}_{f}\left[h_{t-1},x_{t}\right]+b_{f})$$

(7) $$o_{t}={\sigma (w}_{o}\left[h_{t-1},x_{t}\right]+b_{o})$$

Where *i* is the input gate, *f* is the forget gate, *o* is the output gate, *t* is the current timestep, σ is the sigmoid function, *w* is the weight for the gate, *h* is the output of the LSTM network, *x* is the current input, and *b* is the bias for the gate.

The idea behind LSTM is very straightforward: each activation function *c* (called constant error carousel - CEC) is used as a node in a memory cell at timestep *t* and connects to itself with a fixed weight of 1.0. Back propagated errors going through a CEC cannot shrink or grow out of bounds (unless not going through a CEC but to other neural network’s adaptive parts) because of the constant derivative of 1.0 from the function *c*. Nonlinear behaviour can be learnt by different nonlinear adaptive units that are connected to CECs, and some have multiplicative activation functions. Without CECs, previous RNNs had failed to memorise events even only 10 discrete time steps ago, while LSTM neural networks can trace back events that happened thousands of time steps and change the weight accordingly. The CEC *c*, its candidate $\tilde{c}$, and the final output *h* are represented as follows:

(8) $${\tilde{c}}_{t}={\tanh(w}_{c}\left[h_{t-1},x_{t}\right]+b_{c})$$

(9) $$c_{t}=f_{t}*c_{t-1}+i_{t}*\;{\tilde{c}}_{t}$$

(10) $$h_{t}=o_{t}*\tanh\left(c^{t}\right)$$

LSTM can also be applied in many different variants and topologies that use modifiable CECs with self-connections.^38,39^

Positives: LSTMs address the vanishing gradient issue by ignoring some unimportant data within the network. Moreover, LSTMs require no fine-tuning since they provide valuable parameters such as learning rate and input/output gate biases. Also, LSTMs reduce the complexity of updating each weight to *O*(1), which is minimal compared to other approaches

Negatives: LSTMs take much longer time and memory to train and hence require a lot of hardware resources and memory bandwidths. LSTMs are also prone to overfitting, and dropout is much harder to implement in LSTMs. Finally, LSTMs are easily affected by different random weight initialisations.

## Method

### Virtual environment and haptic-based collaborative task

**Figure 1.**
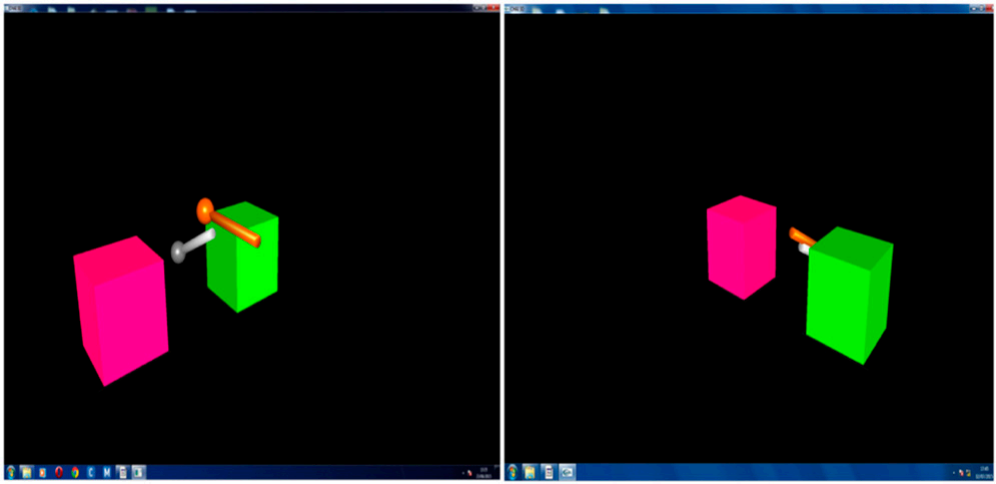
Virtual environment for the task. Each participant has different viewpoint. Participants can control virtual styluses by interacting with the Phantom Omni robots to stack one of the cubes to the top of the other one.

Twenty-four naive healthy participants were recruited (mean age: 26.36, standard deviation: 5.76; gender: eight females and 16 males). They worked in pairs (randomly formed) to lift cubes in a shared virtual environment and stack them on top of each other (Figure 1). All pairs and participants were then numbered from 1 to 12 and 1 to 24, respectively (e.g., pair one includes participant one and 2).

**Figure 2.**
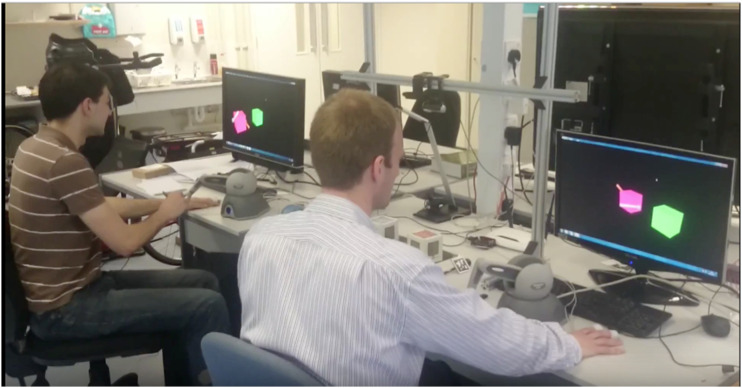
Real-world setup of participants doing the task. They were allowed to talk to each other while competing the task.

Participants performed the task using a Phantom Omni (Figure 2) – a robotic interface with haptic feedback – and were allowed to talk to each other to complete the task successfully.^40^ Participants were instructed to collaborate using the Phantom Omni haptic devices to control their virtual styluses to stack the cubes on top of each other (see Figures 1 and 2).

Position, orientation and force data were collected during their interactions while performing the task. The data was collected at every frame of the application, and since the application was running at 60 frames per second (fixed framerate), there were 60 data points recorded per second. The position data recorded was x, y, and z linear acceleration. The force data was the magnitude of the interaction force in Newton. The orientation data was x, and z Euler angle; all data were fitted into fixed windows of 2.13 s (128 timesteps) for the network training.

All participants were provided written informed consent, and this study was approved by Middlesex University research ethics committee on January 21st, 2015.

### Training

**Figure 3.**
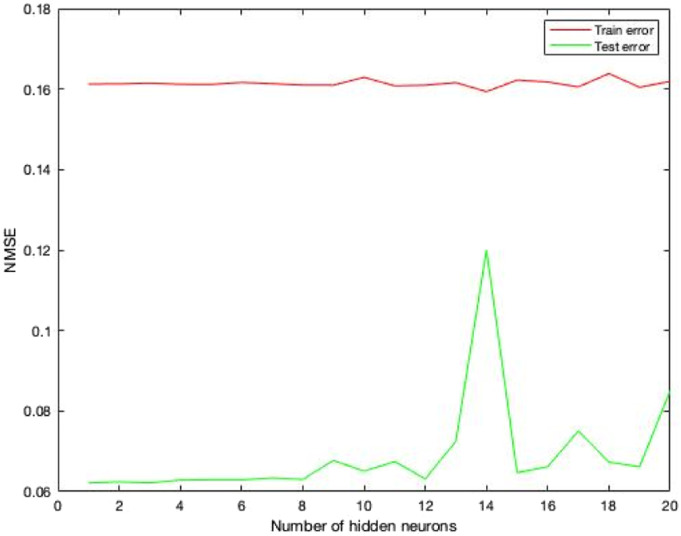
ANN simulation of interaction forces to determine the optimal number of hidden neurons Red line represents the training error while using datasets to train the ANN while green line is the test error of the performance from trained ANN.

Twelve datasets were recorded, but only nine datasets were used due to missing data from three datasets (pairs 1, 2 and 11 failed to complete the task; hence data collected was insufficient for network training). Therefore, eight datasets (pairs 3–10) have been used to train the networks, and one dataset (pair 12) has been used for testing. The first participant’s data from each pair was used for the input, while the second participant’s data was the output (e.g., in pair 3, participant 5’s data was the input and participant 6’s data was the output). The predictor can predict the virtual stylus’s interaction forces, positions, and orientations from one participant based on their partner’s inputs. The error of this training process is called training error, as shown in Figure 3. The estimation from this predictor could help to maintain the smoothness of the interaction via a high-latency network condition. A simulation has been performed to determine the optimal number of hidden neurons to optimise the training. There was one predictor created for each number of hidden neurons (from 1 to 20); the error from the training process was called train error, and the performance of each predictor was tested using the remaining dataset with the error called test error in Figure 3.

The predictor’s performance was determined by calculating the normalised mean squared errors (NMSE). To have a better result in the real-time experiment, the value of test error should be minimum.^41,42^ As shown in Figure 3, when the number of hidden neurons was over 12, the training was over-fitted as the test error increased significantly. Thus, the predictor trained with 12 hidden neurons was selected.

## Results

**Figure 4.**
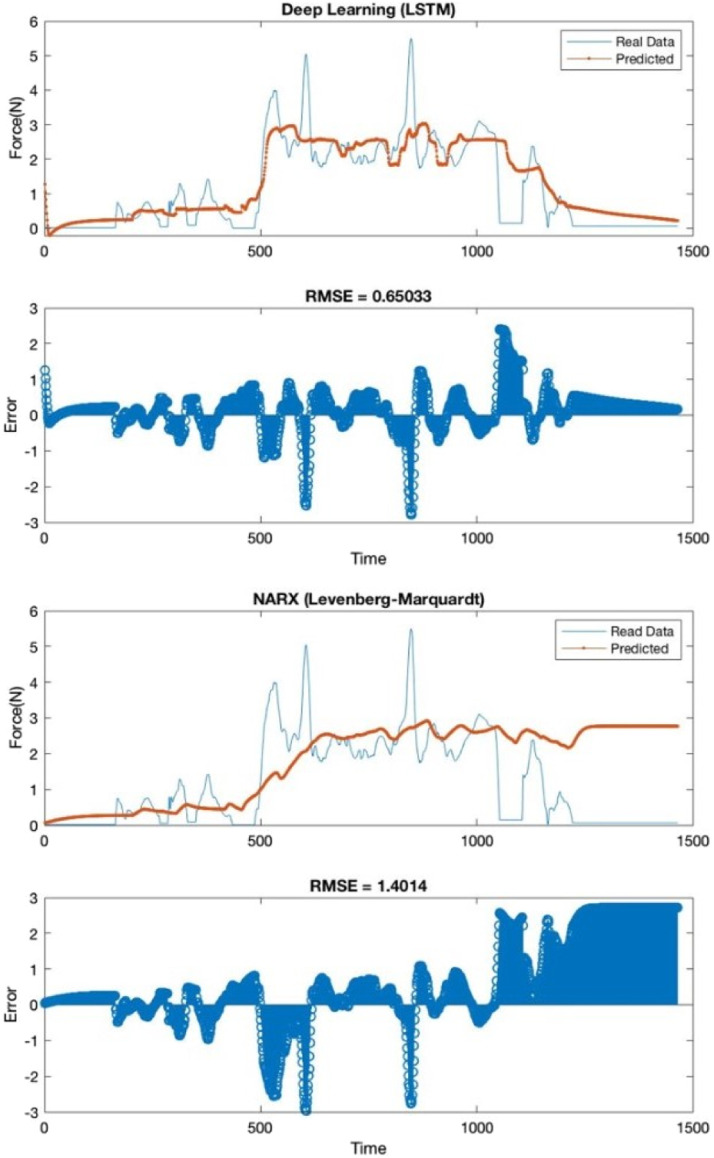
Forces estimation in Newton using LSTM (top chart) and NARX (bottom chart). RMSE is the root mean square error. The blue line is the real data collected from the participant, and the red line is predicted data generated from the AI. The top plot shows a significantly lower error (different from real data) than the bottom plot (53.57% less).

**Figure 5.**
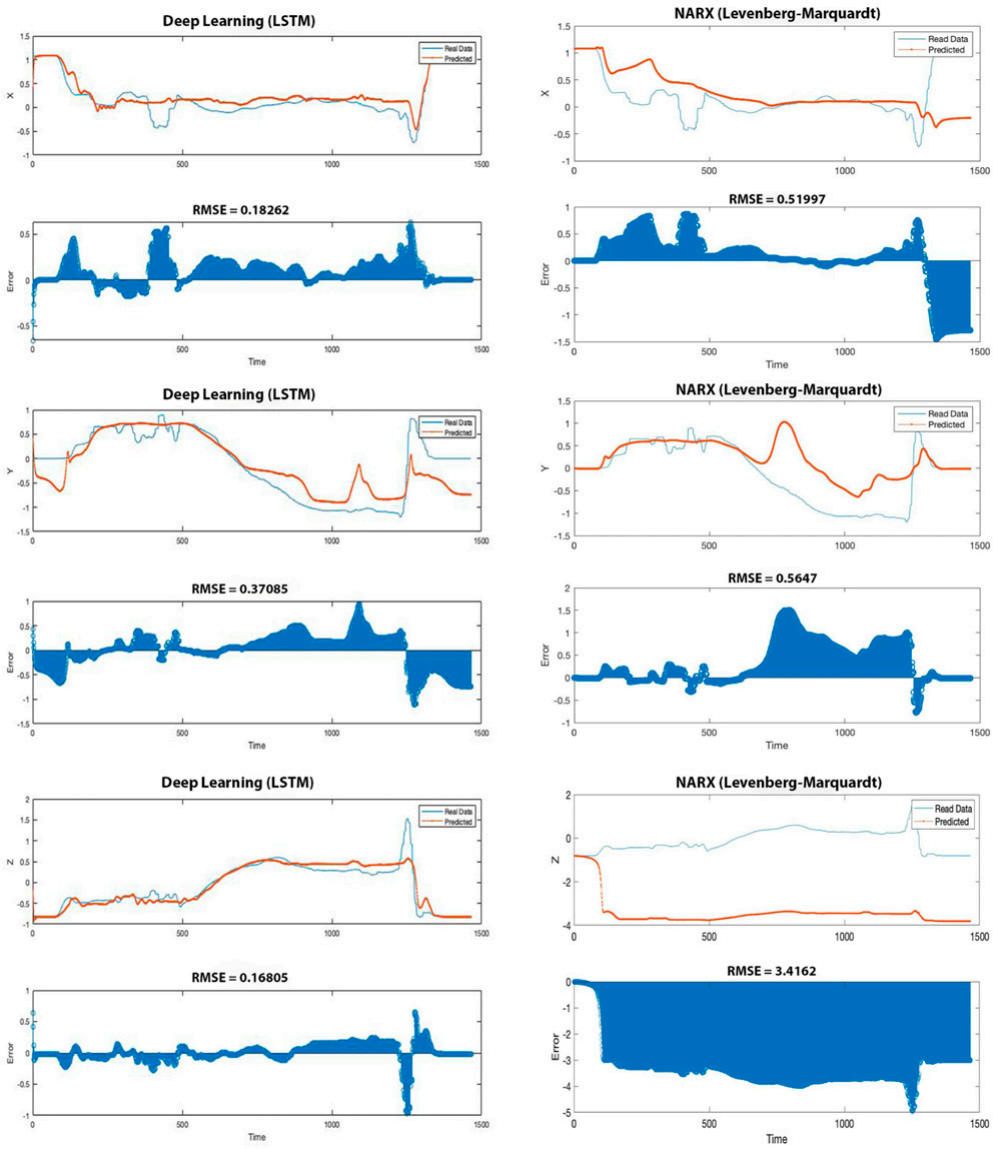
Position estimation using LSTM (left column) and NARX (right column). RMSE is root mean square error. Blue line is the real data collected from participant and the red line is predicted data generated from the AI. The left column plots show significantly lower error (different from real data) than the right column plots (65.38% less in X positions, 33.93% less in Y positions and 95% less in Z positions).

**Figure 6.**
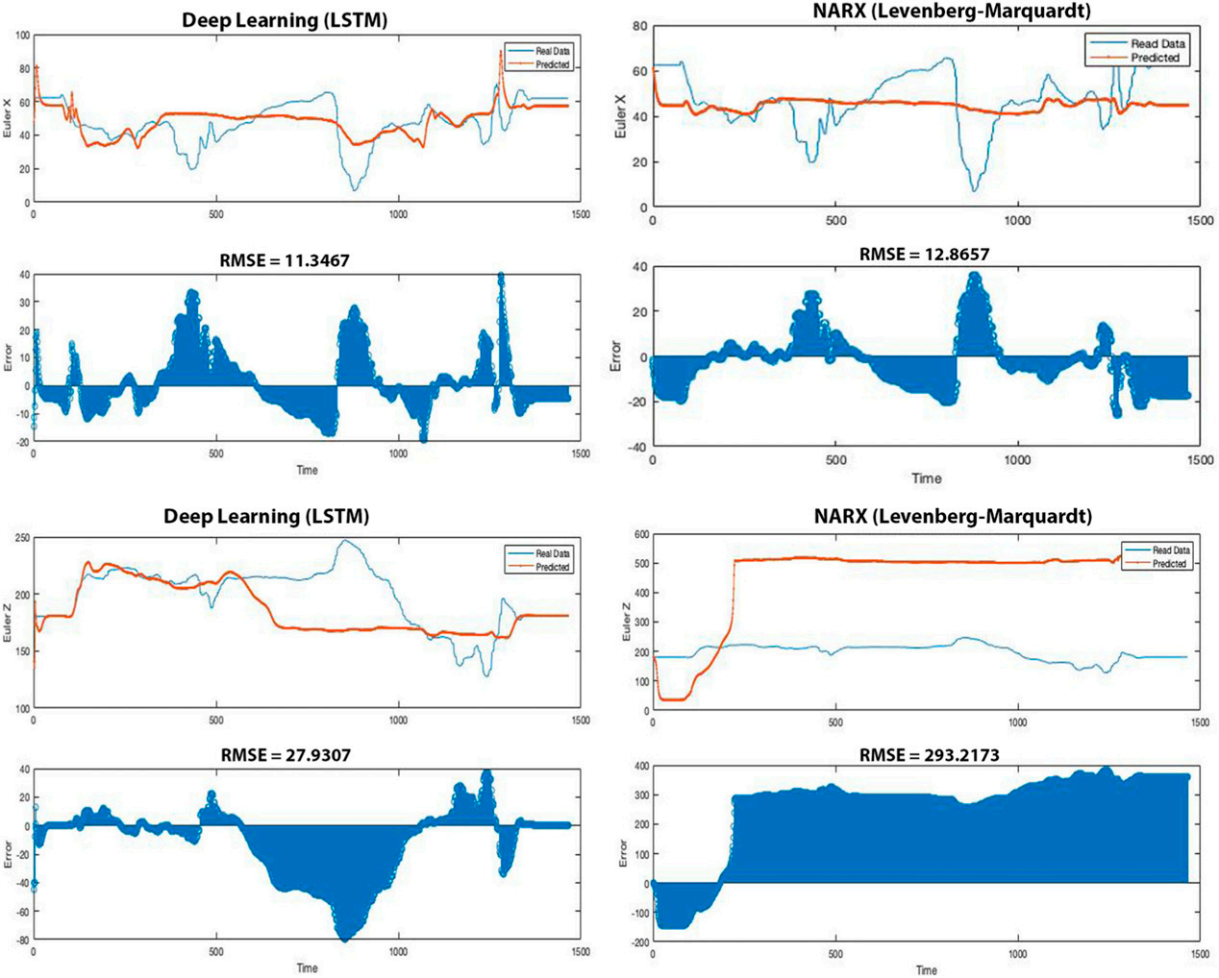
Orientation estimation using LSTM (left column) and NARX (right column). RMSE is root mean square error. Blue line is the real data collected from participant and the red line is predicted data generated from the AI. The left column plots show significantly lower error (different from real data) than the right column plots (11.72% less in Euler X, 90.48% less in Euler Z).

Figures 4, 5 and 6 show the estimated data (forces applied, positions, and orientations of the virtual stylus) generated by the predictor (trained by two different methods) versus the real data collected from the participants (dataset of pair 12).

**Figure 7.**
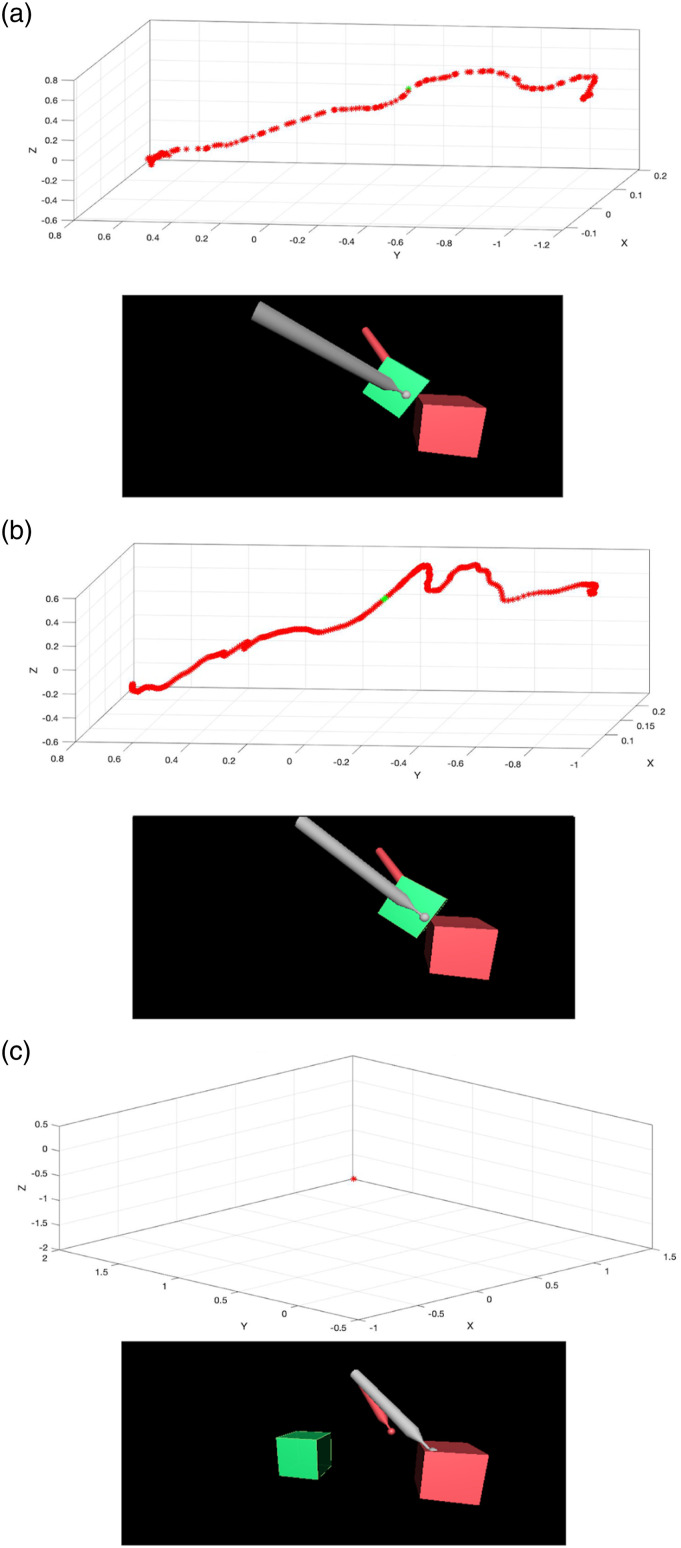
The cube’s trajectories in 3D space (top figures – trajectories of the cube from start to finish) and 3D representations of the objects and tools (bottom figures, halfway until completion of the task, marked as green points in top figures). (A) Real data. (B) Estimation from LSTM, completed the task successfully. (C) Estimation from NARX, failed to complete the task hence there was no trajectory for the cube.

Figure 7 shows the cube’s trajectories in 3D space and 3D representations of objects and tools from real data and estimations from LSTM and NARX methods.

The orientations of the virtual tool were recorded in Euler angles. In Unity – the software used to simulate the virtual environment and collect data – Euler angles are determined by the rotations performed around individual axes: The Z axis, the *X* axis, and finally, the *Y* axis. Because in this study, there was no roll movement of the tool performed, the rotation of the *Y* axis was fixed, and no orientation data was recorded for this axis.

Table 1 shows the errors on each value tested from LSTM and NARX methods. It clearly states that when changing the method from NARX to LSTM, the errors were significantly reduced, indicating that the LSTM method was much more accurate in predicting user’s interaction for this task than NARX.

**Table 1.** Comparison of RMSE results on the values of LSTM and NARX methods.

Values	NARX	LSTM	Reduced
Forces	F = 1.40	F = 0.65	−0.75 (−53.57%)
Positions	X = 0.52	X = 0.18	−0.34 (−65.38%)
	Y = 0.56	Y = 0.37	−0.19 (−33.93%)
	Z = 3.40	Z = 0.17	−3.23 (−95%)
			Avg. −64.77%
Orientations	X = 12.8	X = 11.3	−1.5 (−11.72%)
	Z = 293.2	Z = 27.9	−265.3 (−90.48%)
			Avg. −51.1%

Note. Reduced: Reduced errors when changing NARX to LSTM.

Overall, the results showed that deep learning with the LSTM algorithm was significantly better (53.57% better in forces, average 64.77% better in positions and 51.1% better in orientation) than NARX with the LM algorithm. Data generated from NARX network was not enough to complete the task successfully (force applied and position/orientation from AI agent were not accurate enough to help the human agent), while deep learning with LSTM showed the results that were very close to human interactions (was able to fulfil the task successfully within 25 s against 23 s as seen in real data).

## discussion

Results from LSTM show a great potential to predict user’s behaviour to reduce the negative effects of network delay. A simulation was run in Unity 3D with the LSTM algorithm as a predictor. The remaining dataset collected from the participants (completely different from the eight datasets used for training) has been used as the test data. In this dataset (pair 12), the first participant’s data was used as input, while the AI agent replaced the second participant.

The results of the NARX network may be improved by applying a different learning algorithm. The LM algorithm used in this paper might not be suitable for this particular task.

Regarding the LSTM network, the task could be finished successfully even though the data from one user was completely missing (Figure 7(b) shows that the estimation from LSTM can fulfil the task. Thus, the cube’s trajectory in 3D space was recorded). This result suggests a new approach: all haptic data can be rendered locally to reduce the amount of data being transmitted via the network, while a predictor can help to compensate for the user’s input loss. This approach would take advantage of methods 1 and 2, as mentioned in section II.A.

The cooperative task introduced in this paper can fit the cooperative bilateral interaction for telerehabilitation, as mentioned in the introduction. Therapeutic exercises for rehabilitation are usually repetitive and have a specific goal (e.g., moving an object or reaching a shelf) that is similar to this particular task, thus making this method a possibility for use with those exercises

It is also worth mentioning that when two participants were working on this collaborative task, they had to communicate to each other to come up with a consensus strategy to fulfil the task. The predictor has completely removed this requirement since the algorithm generates all the input data from one participant. As a result, it has made the task easier to complete and eliminated the social interaction between two participants. Hence, the predictor should only be used as a last resort to support the telerehabilitation system when the network condition is poor.

## Conclusion

This paper compares the predictor’s performances from two well-known but distinctive algorithms for a collaborative task in a virtual environment via a network connection. The simulation results from this paper suggest that applying an appropriate algorithm can help the predictor complete the task successfully. Furthermore, this method could be beneficial to apply to similar existing therapeutic exercises that require haptic feedback, thus making telerehabilitation available despite the network condition.

A suggestion for future work is to test this predictor in real time. We are devising the predictor module with two modes:

• Active-assisted: this mode will be enabled when the system detects a colossal delay (more than 100 ms) in the network, making it impossible to update the tool’s positions in real-time. The predictor will help by selecting the best solution based on the user’s profile. The subject will need to make the initial move; the system then guesses the contact point between the tool and the object learnt from his or her own historical movements and moves the tool to that contact point. Haptic feedback will be generated correspondingly to match the time frame, i.e., there is no movement correction in this mode; the subject only needs to initialise their movement, and everything else will be generated automatically. Although this does not provide real interaction for the participants, it can still enable meaningful interactions in deplorable network conditions.
• Active: The mode is enabled in a medium delay network condition (latency at 25 ms to 100 ms), e.g., the tool can still be updated in real-time; however, it is not fast enough to have smooth haptic feedback. In this mode, subjects can move their tool freely to choose the contact point. Once a contact point is selected, the system will adjust the subject’s movement by comparing their real-time end-effector’s position and the similar (or closest) one learned before to maximise the natural feel of interaction. The haptic feedback will be produced based on the pre-learned position locally to ensure smoothness.
